# The association between arterial stiffness and tongue manifestations of blood stasis in patients with type 2 diabetes

**DOI:** 10.1186/s12906-016-1308-5

**Published:** 2016-08-27

**Authors:** Po-Chi Hsu, Yu-Chuen Huang, John Y. Chiang, Hen-Hong Chang, Pei-Yung Liao, Lun-Chien Lo

**Affiliations:** 1 Graduate Institute of Chinese Medicine, China Medical University, Taichung, Taiwan; 2 Department of Chinese Medicine, Changhua Christian Hospital, No.135 Nanxiao St., Changhua, Taiwan; 3 School of Chinese Medicine, China Medical University, No. 91, Hsueh-Shih Rd., Taichung, Taiwan; 4 Department of Medical Research, China Medical University Hospital, Taichung, Taiwan; 5 Department of Computer Science and Engineering, National Sun Yat-sen University, Kaohsiung, Taiwan; 6 Department of Healthcare Administration and Medical Informatics, Kaohsiung Medical University, Kaohsiung, Taiwan; 7 Research Center for Chinese Medicine and Acupuncture, China Medical University, Taichung, Taiwan; 8 Department of Endocrinology, Changhua Christian Hospital, No.135 Nanxiao St., Changhua, Taiwan; 9 Graduate Institute of Statistical and informational Science, National Changhua University of Education, Changhua, Taiwan

**Keywords:** Traditional Chinese medicine (TCM), Diabetes mellitus (DM), Tongue diagnosis, Blood stasis, Arterial stiffness,and brachial-ankle pulse wave velocity (ba-PWV)

## Abstract

**Background:**

Diabetes mellitus (DM) is a hypercoagulable state and is associated with highly increased risk of vascular complications. In the theory of traditional Chinese medicine (TCM), these vascular complications are classified as blood stasis. Diagnosis of the tongue plays an important role in TCM; a bluish tongue, petechiae, and engorged sublingual collateral vessels are manifestations of blood stasis. This study aimed to characterize the tongue manifestations of blood stasis and derive a relationship between blood stasis and vascular disorders in patients with type 2 DM.

**Method:**

We conducted a cross-sectional study of 140 patients with type 2 DM, and compared demography, laboratory, physical examination, ankle brachial index(ABI), brachial-ankle pulse wave velocity (ba-PWV), and tongue manifestation datas. An automatic tongue diagnosis system was used to capture tongue images and characterize clinical tongue manifestations.

**Results:**

A bluish or petechiae tongue was assoicated with a significant decrease in high-density lipoprotein level, and bluish tongue was associated with significant increase in blood triglyceride in patients with type 2 DM. On assessing arterial stiffness, patients with a petechiae tongue had a higher ba-PWV for both sides (L:1938.41 ± 469.54 cm/sec v.s.1723.99 ± 302.16, *p* = 0.02; R:1937.28 ± 405.55 v.s.1741.99 ± 325.82, *p* = 0.03).

**Conclusion:**

Blood stasis, particularly a tongue with petechiae, may be associated with arterial stiffness in patients with type 2 DM. Furthermore, tongue diagnosis could detect blood stasis relevant to DM and could serve as a feasible predictor for DM.

## Background

As the global population increases and ages, diabetes mellitus (DM) has become a major public health concern worldwide [1–3]. DM increases the risk for disability and premature death, and imparts a substantial socioeconomic burden due to the micro- and macro-vascular complications [3]. The long-term micro-vascular complications of DM include retinopathy [4], nephropathy, neuropathy and macro-vascular complications. DM is a hypercoagulable state and is associated with an increased risk of ischemic events; it is also associated with accelerated atherothrombosis [5, 6]. Arterial stiffness is closely related to the progression of DM complications [7]. Consequently, patients with DM have shown a 2- to 4-fold greater risk for coronary artery disease and cerebrovascular disease than those without DM [8]. Thus, vascular complications and arterial stiffness due to poor blood circulation should be closely monitored in patients with DM.

Blood stasis is one of the most important pathological concepts in traditional Chinese medicine (TCM) [9]. Blood stasis is characterized as a disorder of blood circulation with hallmarks including extravagated or sluggish blood circulation and viscous or congested blood; all of these hallmarks may contribute to various disease pathologies [10]. Many diseases lead to blood stasis, such as cardiovascular disease, cerebral vascular accidents, and DM [11]. Blood stasis is often accompanied by characteristic symptoms, such as pain in a fixed position, a dark-purple colored face, infraorbital darkness, a bluish tongue, an engorged sublingual varicosis, petechiae tongue, or an astringent pulse [12].

Tongue diagnosis is important in TCM [13]. The tongue is connected to the internal organs through the meridians; thus the conditions of the organs, qi, blood, and bodily fluids, as well as the degree and progression of disease, are manifested in the tongue [14]. Clinically, practitioners observe tongue characteristics, such as tongue color and shape, fur color and thickness, and the amount of saliva, to deduce the primary ailment of a patient [15]. A bluish tongue, petechiae and engorged sublingual collateral vessels are potential tongue manifestations of blood stasis [16]. Tongue diagnosis is helpful in detecting blood stasis of rheumatoid arthritis (RA) and could serve as a feasible predictor of RA [17].

However, to the best of our knowledge, no study has focused on tongue diagnosis in patients with type 2 DM, despite the theoretical and clinical applications. This study aimed to investigate the tongue characteristics of and relationship between blood stasis and vascular disorders in patients with type 2 DM.

## Methods

### Patients

We conducted across-sectional study and recruited patients with type 2 DM from the Department of Chinese Medicine, Changhua Christian Hospital, between January 2012 and December 2013. We excluded patients with type 1 DM or type 2 DM who had cancer. One hundred and forty eligible patients with type 2 DM were enrolled. The purpose, procedures, potential risks, and benefits of the study were thoroughly explained to the patients. The personal details and photographs of the patients were kept confidential, and all participants signed consent for publication. This study was approved by the Institutional Review Board of Changhua Christian Hospital (IRB#:111106).

### Data collection

Patient metadata were collected (i.e., sex, age, weight, height, history of DM, and any micro-vascular complications). Physical examinations included blood pressure, body mass index(BMI), waistline, hipline, foot examination, ankle brachial index (ABI), and brachial-ankle pulse wave velocity (ba-PWV). Ba-PWV is a direct measurement of aortic stiffness and is the gold standard of arterial stiffness measurements [18]. ABI is a non-invasive method that assesses the patency of peripheral occlusive arterial disease [19]. Routine biological blood tests included hemoglobin A1c (HbA1c), fasting blood-glucose level, cholesterol, triglyceride (TG), high-density lipoprotein (HDL), low-density lipoprotein (LDL), creatinine (Cr), glutamate oxaloacetate transaminase (GOT) and glutamate pyruvate transaminase (GPT) levels.

### Tongue photographs and procedures

**Fig. 1:**
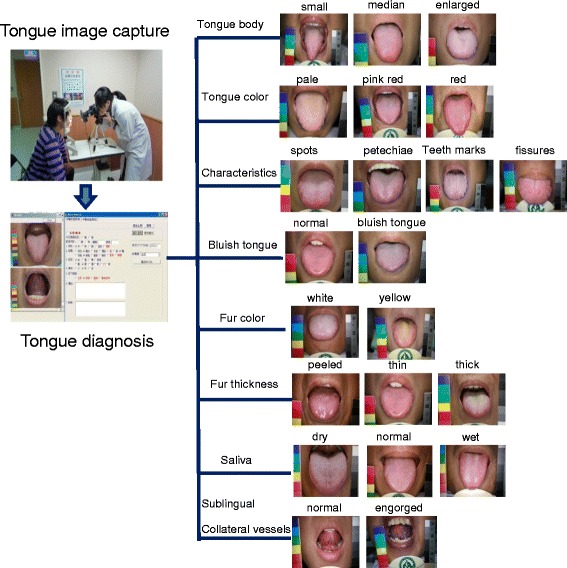
Standard processing flowchart for tongue diagnosis

An automatic tongue diagnosis system was developed to capture tongue images. The consistency and stability of image capturing relies on the brightness and color calibration to compensate for variations, such as intensity and color temperature of light source as well as imaging hardware [15, 16]. Analysis of tongue images was conducted by Chinese medical physicians who had 3–5 years of clinical experience in the Chinese medicine department of Changhua Christian Hospital (CCH), Taiwan. The physicians attended regularly meetings over the past two years and examined over 1000 tongue images from CCH outpatients. Tongue images were identified according to nine primary tongue features: tongue body shape (i.e., small, median, enlarged), tongue body color (i.e., pale, pink red, red), tongue characteristics (i.e., spots, petechiae, teeth-marks, fissures), bluish tongue (i.e., yes, no), fur color (i.e., white, yellow), fur thickness (i.e., peeled, thin, thick), saliva (i.e., dry mouth, normal, wet mouth), and sublingual collateral vessels (i.e., normal, engorged) [15]. Tongue diagnosis followed a standardized protocol (Fig. 1).

### Data analysis

The statistical analysis of datawas performed with IBM SPSS Statistics 19 (IBM Co., New York, NC, USA). After determining if that data was normal (Kolmogorov-Smirnov test), Student’s *t* tests were used to determine differences between continuous variables. Regression models were used to analyze the relationship between the change in ba-PWV and patients possessing tongue manifestations of blood stasis. A *p*-value less than 0.05 was considered statistically significant.

## Result

The characteristics of the 140 patients with type 2 DM were 40 % males (56 patients) and 60 % females (84 patients), the average age was 62.95 ± 11.10 years old (range of 33 to 87 years); the mean BMI was 25.94 ± 3.93 kg/m^2^ , and mean HbA1C was 7.03 ± 1.08 % (Table 1). The duration of DM course in the patients was 2 to 35 years, with an average of 13.57 ± 8.54 years. Twenty-eight (20.0 %) of study patientshad retinopathy; fifty patients (35.7 %) had nephropathy and seventeen patinets (12.1 %) had neuropathy.

**Table 1:** Characteristics of 140 patients with type 2 diabetes

Characteristics	
Age (years, mean ± SD)	62.95 ± 11.10
Gender	
Male (*n*, %)	(56, 40.0 %)
Female (*n*, %)	(84, 60.0 %)
BMI (kg/m^2^, mean ± SD)	25.94 ± 3.93
DM history (years, mean ± SD)	13.57 ± 8.54
≤ 10 years (*n*, %)	(39, 38.6 %)
10 < age ≤ 20 years (n, %)	(42, 41.6 %)
> 20 years (*n*, %)	(20, 19.8 %)
HbA1c (mean ± SD)	7.03 ± 1.08
≤ 7 (*n*, %)	(72, 54.6 %)
7 < HbA1c ≤ 8 (n, %)	(42, 31.8 %)
> 8 (*n*, %)	(18, 13.6 %)
Micro-vascular complication	
Retinopathy (*n*, %)	(28, 20.0 %)
Nephropathy (*n*, %)	(50, 35.7 %)
Neuropathy (*n*, %)	(17, 12.1 %)

Tongue inspection refers to the visual examination of tongue body shape, tongue color, fur color, and fur thickness, as well as other characteristics. The tongue body shape was classified as median (*n* = 123, 87.9 %), enlarged (*n* = 15, 10.7 %), and small (*n* = 2, 2.4 %) (Table 2). The tongue body color was pink red (*n* = 86, 65.2 %), red (*n* = 31, 23.5 %), or pale (*n* = 15 ,11.4 %). Other characteristics observed on the tongue surface included teeth-marks (*n* = 80, 57.1 %), fissures (*n* = 33, 23.6 %), spots (*n* = 32, 22.9 %) and petechiae (*n* = 22,15.7 %). A bluish toned tongue may indicate a problem with blood circulation (*n* = 34, 24.3 %). The classifications of fur color and thickness were white (*n* = 97, 73.5 %) or yellow (*n* = 35, 26.5 %) and thin fur (*n* = 64, 47.1 %), thick fur (*n* = 64, 47.1 %), or peeled fur (*n* = 8, 5.9 %), respectively. The amount of saliva observed was normal (*n* = 110, 80.9 %), dry (*n* = 20, 14.7 %), or wet (*n* = 6, 4.4 %). There were 99 patients (73.3 %) with engorged sublingual collateral vessels.

**Table 2:** Tongue manifestations of patients with type 2 diabetes

Tongue manifestations	Types	*N* (%)
Tongue body	median	123 (87.9 %)
	enlarged	15 (10.7 %)
	small	2 (2.4 %)
Body color	pink red	86 (65.2 %)
	red	31 (23.5 %)
	pale	15 (11.4 %)
Tongue characteristics	spots	32 (22.9 %)
	petechiae	22 (15.7 %)
	teeth-marks	80 (57.1 %)
	fissures	33 (23.6 %)
Bluish tongue	no	106 (75.7 %)
	yes	34 (24.3 %)
Fur color	white	97 (73.5 %)
	yellow	35 (26.5 %)
Fur thickness	thin	64 (47.1 %)
	thick	64 (47.1 %)
	peeled	8 (5.9 %)
Saliva	normal	110 (80.9 %)
	dry mouth	20 (14.7 %)
	wet mouth	6 (4.4 %)
Sublingual collateral vessels	engorged	99 (73.3 %)
	normal	36 (26.7 %)

**Fig. 2:**
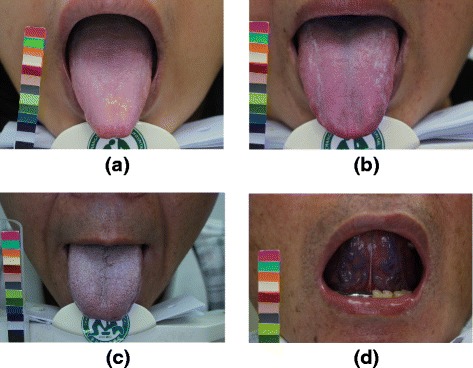
Representative images of tongue manifestations: (**a**) normal tongue; (**b**) petechiae tongue; (**c**) bluish tongue, and; (**d**) engorged sublingual collateral vessels

According to the TCM theory, a bluish tongue, petechiae, and engorged sublingual collateral vessels are potential manifestations of blood stasis (Fig. 2). Therefore, the physical examinations and laboratory data were further examined to address the relationship between vascular disorders and blood stasis related to tongue characteristics (i.e., bluish tongue, petechiae, or engorged sublingual collateral vessels; Table 3). A bluish tongue was correlated with significant decrease in HDL (*p* = 0.03) and a significant increase in TG (*p* = 0.04) in the lipid profile (Table 3). Interestingly, both the left- and right-side ba-PWV (L: 1938.41 ± 469.54 cm/sec v.s. 1723.99 ± 302.16 cm/sec, *p* = 0.02; R: 1937.28 ± 405.55 cm/sec v.s. 1741.99 ± 325.82 cm/sec, *p* = 0.03) were significantly higher in patients with a petechiae tongue than in patients with type 2 DM.

**Table 3:** Comparison of physical examinations and laboratory data of patients with type 2 diabetes with and without tongue manifestations of blood stasis

Variables	Bluish tongue		*p*-value	Petechiae		*p*-value	Engorged sublingual collateral vessels		*p*-value
	Without (*n* = 106)	With (*n* = 34)		Without (*n* = 118)	With (*n* = 22)		Without (*n* = 36)	With (*n* = 99)	
BMI(kg/m^2^)	25.53 ± 3.56	27.01 ± 4.69	0.09	25.85 ± 4.10	26.30 ± 3.23	0.65	26.04 ± 3.25	25.96 ± 4.25	0.94
Waistline(cm)	87.10 ± 9.96	89.50 ± 11.36	0.33	87.13 ± 10.26	89.85 ± 10.55	0.30	87.87 ± 7.90	87.76 ± 11.17	0.97
Hipline(cm)	95.86 ± 7.75	97.84 ± 9.57	0.31	96.33 ± 8.70	96.51 ± 6.49	0.93	96.36 ± 4.43	96.59 ± 9.22	0.88
Systolic pressure(mmHg)	122.44 ± 13.84	129.28 ± 13.39	0.06	123.04 ± 13.96	131.50 ± 11.98	0.10	121.61 ± 13.60	124.18 ± 14.04	0.45
Diastolic pressure(mmHg)	71.44 ± 4.04	73.72 ± 4.86	0.04	71.61 ± 4.24	74.75 ± 3.81	0.05	71.87 ± 4.71	71.79 ± 4.18	0.94
Foot examination score	1.49 ± 1.62	1.26 ± 1.18	0.53	1.41 ± 1.51	1.50 ± 1.54	0.81	1.21 ± 1.35	1.46 ± 1.53	0.51
ABI(L)	1.10 ± 0.11	1.12 ± 0.09	0.36	1.10 ± 0.11	1.15 ± 0.09	0.07	1.06 ± 0.15	1.12 ± 0.09	0.05
ABI(R)	1.13 ± 0.09	1.12 ± 0.12	0.66	1.12 ± 0.11	1.15 ± 0.06	0.17	1.10 ± 0.13	1.13 ± 0.07	0.28
ba-PWV(L)(cm/sec)	1743.10 ± 360.03	1817.88 ± 316.40	0.36	1723.99 ± 302.16	1938.41 ± 469.54	0.02	1725.53 ± 368.85	1761.38 ± 348.82	0.71
ba-PWV(R)(cm/sec)	1760.39 ± 366.31	1831.88 ± 305.58	0.38	1741.99 ± 325.82	1937.28 ± 405.55	0.03	1710.88 ± 383.36	1787.58 ± 346.15	0.43
HbA1c(%)	7.06 ± 1.11	6.94 ± 1.01	0.59	7.05 ± 1.08	6.94 ± 1.13	0.66	7.16 ± 1.33	6.97 ± 0.97	0.38
AC sugar(mg/dl)	137.05 ± 34.33	136.29 ± 25.85	0.91	137.33 ± 33.33	134.41 ± 27.10	0.70	139.09 ± 44.13	135.90 ± 27.37	0.69
Cholesterol(mg/dl)	168.97 ± 34.17	168.03 ± 34.90	0.89	170.01 ± 34.69	162.09 ± 31.57	0.32	168.97 ± 37.21	169.10 ± 33.33	0.98
TG(mg/dl)	116.70 ± 62.92	145.47 ± 86.96	0.04	118.84 ± 67.86	149.95 ± 79.17	0.06	139.74 ± 76.76	119.10 ± 69.06	0.14
HDL(mg/dl)	50.17 ± 13.88	44.32 ± 11.97	0.03	49.90 ± 14.19	42.59 ± 7.88	0.02	47.83 ± 12.89	49.45 ± 14.09	0.55
LDL(mg/dl)	96.05 ± 27.10	91.19 ± 24.12	0.35	95.68 ± 26.37	90.50 ± 26.72	0.40	98.17 ± 29.54	93.59 ± 25.37	0.38
Cr(mg/dl)	0.93 ± 0.50	0.99 ± 0.43	0.56	0.94 ± 0.50	0.96 ± 0.40	0.89	0.90 ± 0.30	0.97 ± 0.54	0.47
GOT(U/L)	27.88 ± 15.69	32.03 ± 21.16	0.30	28.66 ± 17.61	30.32 ± 15.62	0.68	29.22 ± 19.24	28.92 ± 16.99	0.93
GPT(U/L)	27.66 ± 15.76	33.59 ± 31.47	0.30	28.76 ± 20.72	31.09 ± 21.85	0.63	29.79 ± 19.02	29.30 ± 21.90	0.91
Microalbumin(mg/day)	286.79 ± 982.78	374.99 ± 1094.05	0.67	352.04 ± 1096.11	95.14 ± 205.03	0.03	244.97 ± 731.47	339.18 ± 1111.65	0.67

Values represented as mean ± SD. *p*-values performed by independent *t* test

*HbA1c* hemoglobin A1c, *AC sugar* fasting blood-glucose level, *TG* triglyceride, *HDL* High-density lipoprotein, *LDL* low-density lipoprotein, *Cr* Creatinine, *GOT* glutamate oxaloacetate transaminase, *GPT* glutamate pyruvate transaminase, *BMI* body mass index, *ABI* ankle brachial index, *ba-PWV* brachial-ankle pulse wave velocity

## Discussion

The core of assessment in Chinese medicine is “pattern identification/syndrome differentiation and treatment” based on inspection, listening and smelling examinations, inquiry, and palpation. Inspection is the most important of the four assessments, and tongue assessment is a crucial part of observation. Tongue appearance is a crucial indicator of physiological and pathological changes to the internal organs [19]. Studies have shown that tongue diagnosis plays an important role in clinical prognosis of RA and DM [15, 16, 20–22].

To the best of our knowledge, this is the first attempt to apply TCM tongue diagnosis to the survey of patients with type 2 DM. Tongue inspection refers to the shape, color, and fur color, and fur thickness, as well as other characteristics [23]. In patients with DM, buccal alterations can be easily observed with adequate glycemic control. Dry mouth is generally associated with decreased saliva production and is present in 10 to 30 % patients with DM; in these patients, a coated tongue is also observed [24]. In TCM, diabetes-related symptoms are referred to as “Xiaoke”, which means increased thirst (or polydipsia), since as long as 2000 years [25]. Furthermore, we show that 47.1 % of patients possessed a coated tongue (i.e., thick fur). According to the TCM theory, tongue fur indicates the Yang organs, especially the digestive system. Thick fur is usually associated with phlegm-dampness and patterns of blood stasis [26]. Thus, understanding and interpreting these tongue manifestations of DM by TCM are important for both in theoretical and clinical applications.

Pulse wave velocity (PWV) is a noninvasive clinical index of arterial stiffness. Arterial pulse wave velocity reflects the stiffness of arteries, and serves as an indicator of atherosclerosis [27, 28]. Arterial stiffness is an age-related process that is present in numerous diseases, including DM. The PWV of patients with DM is higher than that of healthy subjects [29]. According to previous studies, ba-PWV ≥ 1600 cm/sec is an independent risk factor for cardiovascular disease and vascular complications [30]. Here, we observed an average ba-PWV above 1700 cm/sec in patients with type 2 DM; this implies arterial stiffness.

Tongue manifestations are important features for detecting blood stasis [17]. Our study revealed that the tongue manifestations of blood stasis (i.e., petechiae tongue, bluish tongue, or engorged sublingual collateral vessels) corresponded to higher ba-PWV, particularly for patients with petechiae tongue. Furthermore, we evaluated the relationship between the change of ba-PWV and the number of blood stasis tongue manifestations. The results showed that patients possessing increased blood stasis tongue manifestations had significantly increased mean ba-PWV (78.8 cm/sec; *p* = 0.037). This suggests that patients with type 2 DM have increased blood stasis tongue manifestations that are correlated with severe arterial stiffness.

There were several limitations of our study. First, the sample size was relatively small. Second, we did not enroll healthy controls or patients with type 1 DM; this is because most patients with type 1 DM only use conventional medicine (i.e., insulin injection) and do not utilized TCM as a complementary therapy. Therefore, further studies with larger sample sizes, including healthy subjects as well as type 1 and type 2 DM groups, are required to determine the relationship between tongue manifestations and disease. This proposed study can provide a rationale for a wider use of tongue diagnosis in clinical practice.

## Conclusion

Blood stasis of the tongue, particularly petechiae tongue, is associated with arterial stiffness in patients with type 2 DM. Tongue diagnosis is helpful for detecting blood stasis and could serve as a feasible predictor of DM.

## Abbreviations

ABI: Ankle brachial index
AC sugar: Fasting blood-glucose level
baPWV: Brachial-ankle pulse wave velocity
BMI: Body mass index
CCH: Changhua Christian Hospital
Cr: Creatinine
GOT: Glutamate oxaloacetate transaminase
GPT: Glutamate pyruvate transaminase
HbA1c: Hemoglobin A1c
HDL: High-density lipoprotein
LDL: Low-density lipoprotein
TCM: Traditional Chinese medicine
TG: Triglyceride
